# Editorial: Sex and Gender Aspects in Diabetes

**DOI:** 10.3389/fendo.2019.00813

**Published:** 2019-11-26

**Authors:** Alexandra Kautzky-Willer, Mia von Euler, Sabine Oertelt-Prigione

**Affiliations:** ^1^Gender Medicine Unit, Division of Endocrinology and Metabolism, Department of Internal Medicine III, Medical University of Vienna, Vienna, Austria; ^2^Departments of Clinical Science and Education, Södersjukhuset and Medicine, Karolinska Institutet, Solna, Sweden; ^3^Department of Clinical Pharmacology, Karolinska University Hospital, Huddinge, Sweden; ^4^Department of Primary and Community Care, Radboud University Medical Center, Nijmegen, Netherlands; ^5^Institute of Legal and Forensic Medicine, Charité-Universitätsmedizin, Berlin, Germany

**Keywords:** sex, gender, diabetes, cardiovascular, complications, therapy

Sex differences affect every cell and living organism. Gender impacts all our behaviors, choices, and perceptions as human beings. Both factors, the biological and the psycho-social, are essential mechanisms that shape our interaction with the health care system. They influence access to healthcare, diagnostic paths, therapeutic choices, and long term outcomes. Although the scientific knowledge is constantly increasing, the inclusion of sex and gender in clinical practice and medical research is still limited (1).

## To the Detriment of Our Patients

Sex as a biological variable (SABV) represents an easily accessible and cost-free variable that frequently acts as a disease modulator. Sex differences do, thus, represent the first step toward personalization of healthcare. As demonstrated by John et al. in this special edition, sex differences in cardiac morphology are already visible at the animal model level and do translate into differences described in humans. If overlooked, these differences will affect reproducibility and limit the amount of information gathered from costly and time-consuming experiments. Analyzing sex is sensible from an economic standpoint, because it represents a freely available stratifying variable and because in the spirit of the 3Rs (replace, reduce, refine) we should gather all possible information from animal studies if we chose to perform them.

Next to the obvious ethical and economic implications, the inclusion of SABV also has clear implications for patient safety. Reports of sex differences in response to therapies (2) and in the incidence of unwanted side effects or overt toxicity (3) are accumulating. Next to the consideration in all phases of clinical trials, sex should be investigated as a biological modulator of diagnostic sensitivity. Physiological differences might impact sensitivity of screening tests, such as fecal occult blood screening (FOBS) (4), or glucose screening (5). Potential differences in the effectiveness of troponin as a marker for cardiac injury have also been reported. We need further study to clarify if commonly used clinical markers are efficacious for both sexes as reported by Leutner et al. The authors did not find any sex differences in the predictive ability of both TnI and TnP in a population of 818 patients referred for coronary stenting.

While examining the role of sex and gender, researchers and clinicians will need to take a life course perspective. Male and especially female bodies change along the life course making them more susceptible for disease as hormonal status changes. As Zhang et al. reported in their study women tend to be more at risk for the development of diabetes after menopause, although the overall prevalence of the disease does not differ between women and men in the investigated population in China. The fact that women might develop cardiovascular diseases later in life might require some logistic adaptation in designing cohort studies. Women might have to be observed for longer periods of time in cohort studies and might have to be oversampled if a certain number of outcomes is needed for analysis. The same holds true for research on diseases that are less represented in males.

Next to the strictly biological differences due to sex, gender might play a significant role in disease incidence, presentation, and management. Patients' experiences and health behavior might differ according to gender, as Raparelli et al. demonstrated in their study. Diabetic women, which are at higher risk of cardiovascular disease compared to men, were more likely to display reduced medication adherence than men with ischemic heart disease (IHD).

Gender might also be a significant factor in the design of preventative measures and the uptake of specific therapeutic options. As Harreiter and Kautzky-Willer describe in their review, male diabetics were less likely to participate in preventative physical activity, although gender-homogeneous groups represented an advantage compared to mixed groups. Women on the other hand, were more likely to be referred for bariatric surgery. These differences might be associated with gender stereotypes and gender-specific behavioral patterns, which need to be further investigated. The investigation of gender has long been hampered by the lack of appropriate instruments, yet in recent years some progress has been made (6) and currently several studies are developing new questionnaires to investigate the subject.

Overall, the current collection gives a small glimpse of the breadth of topics and the clinical relevance of sex and gender differences in diabetes research. Next steps toward implementation of the subject include the systematic evaluation of sex differences in diagnostic approaches as well as the evaluation of the potential need for sex-specific therapy. Gender aspects, ranging from awareness to preventative behavior and access to healthcare, also need to be addressed. Sex is a readily available variable that can be easily incorporated into analyses, potentially improving their predictive abilities. We owe the best possible care to our patients and as such, this option can no longer be ignored.

## Author Contributions

AK-W, ME, and SO-P fulfill the standard requirements for authorship and have read and approved the submission of the final draft.

### Conflict of Interest

The authors declare that the research was conducted in the absence of any commercial or financial relationships that could be construed as a potential conflict of interest.
